# Shear-Wave Elastography Variability Analysis and Relation with Kidney Allograft Dysfunction: A Single-Center Study

**DOI:** 10.3390/diagnostics10010041

**Published:** 2020-01-13

**Authors:** Sorana D. Bolboacă, Florin Ioan Elec, Alina Daciana Elec, Adriana Milena Muntean, Mihai Adrian Socaciu, Gheorghita Iacob, Răzvan Zaro, Alexandra-Ioana Andrieș, Ramona Maria Bădulescu, Radu Mihai Ignat, Mihaela Iancu, Radu Ion Badea

**Affiliations:** 1 Department of Medical Informatics and Biostatistics, Iuliu Hațieganu University of Medicine and Pharmacy Cluj-Napoca, Louis Pasteur Str., no. 6, 400349 Cluj-Napoca, Romania; miancu@umfcluj.ro; 2 Department of Urology, Iuliu Hațieganu University of Medicine and Pharmacy Cluj-Napoca, Clinicilor Str., no. 4-6, 400006 Cluj-Napoca, Romania; ramona.contis@gmail.com; 3 Department of Renal Transplantation, Clinical Institute of Urology and Renal Transplantation, Clinicilor Str., no. 4-6, 400006 Cluj-Napoca, Romania; dralinaelec@gmail.com (A.D.E.); munteana2@yahoo.com (A.M.M.); iacob.gheorghita@gmail.com (G.I.); 4 Department of Medical Imaging, Iuliu Hațieganu University of Medicine and Pharmacy Cluj-Napoca, Croitorilor Str., no. 19-23, 400162 Cluj-Napoca, Romania; mihaiaso@gmail.com (M.A.S.); Razvan.Zaro@umfcluj.ro (R.Z.);; 5 Department of Medical Imaging, “Prof. Dr. Octavian Fodor” Regional Institute of Gastroenterology and Hepatology, Constanța Str., no. 5, 400162 Cluj-Napoca, Romania; 6 Department of Anatomy, Iuliu Hațieganu University of Medicine and Pharmacy Cluj-Napoca, Clinicilor Str., no. 3-5, 400006 Cluj-Napoca, Romania; ignat.radu@umfcluj.ro

**Keywords:** kidney stiffness, kidney transplantation, Shear Wave Elastography (SWE), allograft dysfunction, estimated Glomerular Filtration Rate (eGFR), ultrasound

## Abstract

Shear-wave elastography (SWE) showed the absence or presence of significant differences among stable kidney allograft function and allograft dysfunction. We evaluated the variability of kidney allograft stiffness in relation to allograft dysfunction, respectively, in terms of a correlation of stiffness with patients’ characteristics. A single-center prospective study on patients who had undergone renal transplantation was conducted between October 2017 and November 2018. Patients were clinically classified as having a stable allograft function or allograft dysfunction. SWE examinations performed by the same radiologist with a LOGIQ E9 were evaluated. Ten measurements were done for Young’s modulus (kPa) at the level of allograft cortex and another ten at the level of medulla. Eighty-three SWE examinations from 63 patients, 69 stable allografts, and 14 allografts with dysfunction were included in the analysis. The intra-examinations stiffness showed high variability, with the quantile covariation coefficient ranging from 2.21% to 45.04%. The inter-examinations stiffness showed heterogeneity (from 28.66% to 42.38%). The kidney allograft cortex stiffness showed significantly higher values in cases with dysfunction (median = 28.70 kPa, interquartile range (IQR) = (25.68–31.98) kPa) as compared to those with stable function (median = 20.99 kPa, interquartile range = (16.08–27.68) kPa; *p*-value = 0.0142). Allograft tissue stiffness (both cortex and medulla) was significantly negatively correlated with body mass index (−0.44, *p*-value < 0.0001 for allograft cortex and −0.42, *p*-value = 0.0001 for allograft medulla), and positively correlated with Proteinuria/Creatinuria ratio (0.33, *p*-value = 0.0021 for allograft cortex and 0.28, *p*-value = 0.0105 for allograft medulla) but remained statistically significant only in cases with stable function. The cortical tissue stiffness proved significantly higher values for patients with allograft dysfunction as compared to patients with stable function, but to evolve as an additional tool for the evaluation of patients with a kidney transplant and to change the clinical practice, more extensive studies are needed.

## 1. Introduction

The kidney is one of the most frequently transplanted organs with 58.9% kidney transplants in the United States of America (USA) from 1988 to 2019 [1]. An increase in kidney transplantation has also been observed in most European Union member states, from 36.8 pmp (performed kidney transplantations per million people) in 2011 to 38.1 pmp 2016 [2]. Furthermore, a slight increase in the kidney transplantation rate is also observed in all non-EU member states, from 11.3 pmp in 2011 to 17.8 pmp in 2016 [2].

Ultrasonography (US) is one imaging method used for the evaluation and follow-up monitoring of kidney allografts [3]. The grey scale US examination is mainly used to evaluate the kidney allograft (position, dimensions, and presence of masses) [4,5]. The kidney transplant vascularity is evaluated by Doppler examination, aimed to identify artery stenosis, thrombosis, dissection, or post-biopsy arterio-venous fistulae [6]. However, the resistivity index (RI) is influenced by extra-renal factors and has low sensitivity and low specificity in the identification of acute renal allograft rejection [7]. Contrast-Enhanced Ultrasonography (CEUS) assesses the microvascular perfusion and is used to identify acute vascular complications and renal graft dysfunction [8]. Real-time elastography (RTE), transient elastography (TE), or acoustic radiation force impulse (ARFI) elastography are used to evaluate renal allograft fibrosis [4,5,9].

The Young’s modulus (YM), expressed in kilopascals (kPa), is a measure of elasticity and showed significantly increased values among patients with chronic kidney diseases (CKD) as compared to healthy controls (median = 9.40 kPa, IQR (inter-quartile range) = (5.55–22.35) kPa vs. median = 4.40 kPa, IQR = (3.68–5.70) kPa; *p*-value = 0.002) [10]. Samir et al. [10] also reported a high intra-subject and inter-measurement YM variability in chronic kidney disease (4.27 kPa, IQR = (2.89–9.90) vs. 1.51 kPa, IQR = (1.21–2.05) kPa; *p*-value < 0.001). Furthermore, statistically higher YM values were observed in women as compared to men (*p*-value = 0.04), among patients with CKD as compared to controls (*p*-value = 0.0002), and in Caucasians as compared to others among healthy controls (*p*-value = 0.01) [10].

The utility of elastography in the assessment of renal parenchyma had been previously reported, but the results are contradictory [11,12,13]. Syversveen et al. [14,15] reported no differences in shear-wave elastography (SWE) values between different grades of kidney allograft fibrosis. However, other authors reported either a significant positive [16] or negative [17] correlation between mean Acoustic Radiation Force Impulse (ARFI) and the grade of fibrosis. Ren et al. reported a higher elasticity expressed as shear wave velocity in the transplanted kidney cortex as compared to the medulla and sinus [18]. Early et al. also demonstrated a statistically significant median medulla SWE associated with renal allograft fibrosis (*p*-value = 0.04), but no significant association with the mean or median cortical SWE values (*p*-value = 0.37) [19]. Lee et al. [20] reported an increase in the mean SWE during the first post-transplantation year. Early et al. summarized personal experience in the SWE evaluation of patients with kidney transplant and listed the following factors that affect the SWE measurements: anisotropy (increased or decreased SWE according to the orientation of medullary pyramids reported to the US beam), urinary pressure (increases SWE, urine pressure determine compression of renal parenchyma), hydronephrosis (increases SWE), vascular occlusions (arterial stenosis decreases SWE by hypoperfusion, venous stenosis increases SWE due to the increased parenchymal pressure), and body mass index (decrease SWE, multifactorial mechanism) [21]. Ghonge et al. evaluated the role of SWE in the differentiation of stable renal allograft from acute allograft dysfunction on a sample of 72 patients (stable function in 30 patients, acute dysfunction in 19 patients and chronic dysfunction in 11 patients) and showed significantly higher parenchymal stiffness on patients with chronic allograft dysfunction (stiffness values in stable function < stiffness values in acute allograft dysfunction < stiffness values in chronic allograft dysfunction (*p*-value, *p* < 0.02) [22]. Ma et al. reported a median stiffness at the cortex of 22.3 kPa (IQR = (19.0–26.5) kPa) and at the medulla of 15.0 kPa (IQR = (13.7–18.0) kPa) in patients with kidney allograft tubulointerstitial fibrosis and a significant correlation between semiquantitative Banff ci/ct score and cortical (*p*-value = 0.004) and medullary (*p*-value < 0.001) tissue stiffness [23]. Despite the advantages provided by the fast diagnosis and non-invasiveness of the SWE technique, several disadvantages were identified [24]: non-specificity of the SWE values among different grades of kidney allograft fibrosis, high impact of transducer pressure on cortical stiffness, high inter- and intra-observed variation, specific software, and hardware requirements.

In the framework of the state-of-the-art, the primary objective of our study was to evaluate the variability of kidney allograft elasticity measured by shear-wave elastography and the relation with clinical and histopathological allograft dysfunction. The secondary objective was to assess if any associations exist between allograft stiffness and patients’ characteristics (e.g., body mass index, time from a kidney transplant to examination, grey-scale ultrasound features, resistivity index, serum creatinine, estimated glomerular filtration rate, and Proteinuria/Creatinuria ratio).

## 2. Materials and Methods

### 2.1. Design of Experiment

This single-center prospective study was approved by the institutional review board (Ethical Committee of the “Iuliu Hațieganu” University of Medicine and Pharmacy Cluj-Napoca, approval number: 1409/19.10.2017), and written informed consent was obtained from all patients. All patients with kidney transplantation evaluated at the Clinical Institute of Urology and Renal Transplantation (CIURT), Cluj-Napoca were eligible to participate in the study. CIURT is a regional renal transplant center in Romania with national addressability and a waiting list of over 2000 CKD stage V patients. The transplant activity started in 1992 in partnership with Interuniversity Organ Transplant Consortium, Rome, and more than 2100 renal transplants were already made, with a maximum annual rate of 168 renal transplants in 2013.

**Figure 1:**
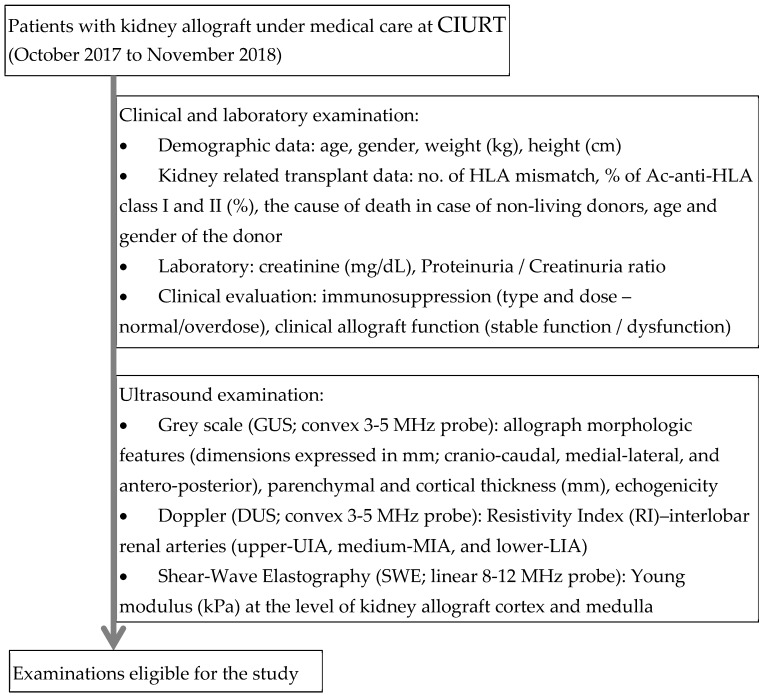
Study design flow: clinical and ultrasound examinations.

Patients over 18 years old who underwent a kidney transplant since 2007 who came for a general health checkup in the period between 1 October 2017 to 30 November 2018 were invited to participate. Patients unwilling to participate, with severe pulmonary hypertension, uncontrolled hypertension, respiratory distress syndrome, with pre-renal or post-renal graft dysfunction, left-to-left shunt, or kidney stones were excluded from the study but received standard medical care. The flow of evaluation and examinations is given in Figure 1.

### 2.2. Clinical Evaluation

The eligible patients were clinically evaluated by a nephrologist who assessed the general health of the patients and recommend laboratory tests (e.g., serum creatinine, Proteinuria/Creatinuria ratio, plasma levels of recommended immunosuppression medication, etc.). Regardless of the blood creatinine value, stable allograft function was considered for patients without changes in the creatinine level as compared to a previous routine health checkup. Allograft dysfunction was clinically considered when an absolute increase in serum creatinine of ≥ 0.3 mg/dL, a percentage increase in serum creatinine of ≥ 50% (1.5-fold from baseline), a reduction in urine output (documented oliguria of less than 0.5 mL/kg/h for more than 6 h) [25], or proteinuria > 1 g/day occurred [26]. Kidney related transplant data were collected from medical charts after the inclusion of patients in the study.

Several variables were calculated from the collected raw data. The time from transplant expressed in days was calculated as the difference between examination and transplantation date (dd/mm/yyyy). Body mass index (BMI) was calculated by the division of mass (kg) to height (m^2^) [27] and patients were classified as underweight (BMI< 18.5 kg/m^2^), normal (18.5 ≤ BMI < 25 kg/m^2^), overweight (25 ≤ BMI < 30 kg/m^2^) or obese (BMI≥ 30 kg/m^2^). The estimated glomerular filtration rate (eGFR, expressed as mL/min/1.73m^2^) was calculated using the formula reported by Levey et al. [28].

The biopsy was recommended by the nephrologist in all cases when clinical allograft dysfunction was observed whenever no previous biopsy was available. The result of the histopathological exam was considered as the gold standard when a biopsy examination existed before the shear wave elastography examination and no clinical changes were observed. One unblinded physician performed the histopathological examinations. Renal biopsy was performed with 18G biopsy needles under ultrasound control, providing two bioptic cores, and the tissue was embedded in paraffin. The paraffin blocks were cut in 3 μm slices and stained with hematoxylin-eosin, periodic acid-Schiff (PAS), trichrome Masson stain, and methenamine silver stains. Immunohistochemistry staining was performed for C4d. BK virus immunohistochemistry was performed when indicated. The pathological report was formulated following the updated 2015 Banff classification [29].

### 2.3. Grey Scale and Doppler Examination

Patients who agreed to participate in the study were referred to ultrasound evaluation, including grey-scale ultrasound, to evaluate the morphology of the kidney allograft, Doppler examination, and SWE (Figure 1). The physician who performed the ultrasounds was blinded to the patient data. Each patient received first a grey scale ultrasound examination followed by a Doppler examination. Kidney dimensions, parenchymal and cortical thickness, and subjective evaluation of echogenicity were recorded for each examination. The kidney allograft volume, expressed in ml, was calculated based on US grayscale measurements using the following corrected ellipsoidal formula: volume = height × width × depth × 0.674 [30]. The resistivity index (RI) at the level of interlobar renal arteries were also measured (Figure 1).

### 2.4. Shear Wave Elastography Examination

The SWE examination was performed with a linear 8–12 MHz probe on a patient with an empty bladder, in the supine position, lying still during the examination, and instructed to breathe normally throughout data acquisition. The linear 8–12 MHz probe is able to more accurately measure surface details (kidney allograft is located more superficial as compared to the native kidney) and thus provide a higher precision elastography measurements (higher frequency is equivalent with more precise measurements of the shear wave speeds). The SWE examination uses dynamic excitation to generate shear waves (SWs) in the body, inducing higher energy ultrasonographic pulses that produce transverse shear rates [31]. The SWs are monitored as they travel in the tissue using a real-time imaging modality. Under the assumption that shear wave speed in a medium is related to the Young’s Modulus (YM, a measure of stiffness), the estimation of shear wave speed is automatically converted to YM. The LOGIQ E9 allows quantitative measurement of stiffness as shear wave speed (m/s) or YM (kPa, kilopascals) in a defined region of interest (ROI) and can be represented on a color-coded map.

**Figure 2:**
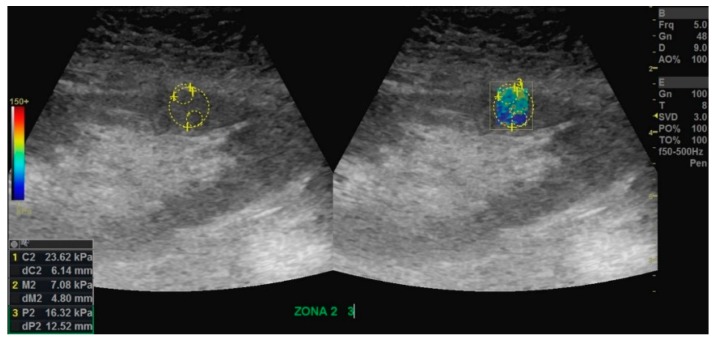
Image from shear-wave elastography shows measurements of stable function kidney allograft on an overweight woman, age 55 years at transplant, examined at two years post-transplant. The image in the left illustrates the renal cortex ROI (1), renal medulla ROI (2), and overall ROI (incorporating renal cortex and medulla, (3). An elastogram box filling with heterogeneous color distribution in the range of low and medium stiffness is observed in the right image (the blue color indicates shiftiness while the red pattern suggests an increased rigidity).

The YM values were obtained using LOGIQ E9 software at the post-processing phase for all patients. The measurements were performed by the same radiologist who individually selected the ROI using the circular tool (around 1 cm circumference, Figure 2). A B-mode kidney allograft image with a clear as possible delimitation between cortex and medulla was used to determine the position of ROI. Three mean measurements for each ROI were collected, namely the cortex, medulla, and thickness of the parenchyma (cortex and medulla), but only the YM on the kidney allograft cortex and medulla were evaluated. Ten cortical and ten medullary ROI measurements obtained on two zones (five ROIs per zone) with similar anisotropy were evaluated. The values of YM were automatically calculated for each ROI by the LOGIQ E9 software for each SWE examination.

### 2.5. Statistical Analysis

Statistica software (version 13.5, StatSoft, Tulsa, OK, USA) and the “cvcqv” and “psy” R packages (version 3.6.1, the R Foundation for Statistical Computing, Vienna, Austria) were performed for statistical analysis. The significance level was set to 5%, and all *p*-values less than 0.05 were considered statistically significant.

The statistical unit in this study was the SWE examination, defined as an agreement of the patient to participate, available clinical and measurements data, ultrasound examination performed by the same radiologist with LOGIQ E9 system (GE’s, General Electric (GE) Healthcare, Wauwatosa, WI, USA). Only examinations that simultaneously achieved all the above criteria were evaluated.

The SWE examination is very sensitive to the pressure of the probe on the examined tissues, so the value of the mean of the ten measurements could be misleading, so the median values were used for comparison between groups and in association analysis. The coefficient of quartile variation (CQV) and the intraclass correlation coefficient (ICC) were used as measures of YMs variability [32]. The CQV was calculated per examination as a measure of intra-examination precision (intra-patient variability) and inter-examinations as a measure of inter-patient variability. The inter-examinations CQV is expressed as a point estimate and associated adjusted bootstrap percentile (BCa) 95% confidence interval [33] with a number of bootstrap replications fixed at R = 10,000.

Qualitative data were reported as numbers and percentages, and according to the expected values, the Chi-square test or the Fisher’s exact test were used to compare frequencies on different groups. Quantitative data were first tested for normality (Kolmogorov–Smirnov test) and since proved not to follow the normal distribution (*p*-values < 0.05) the values were reported as median with interquartile range (IQR = (Q1–Q3), where Q1 is the first quartile and Q3 is the third quartile) and comparisons were done with the Mann–Whitney test (e.g., age, body mass index, time from transplant to SWE examination, serum creatinine level, Proteinuria/Creatinuria ratio, eGRF, Allograft volume, Parenchymal thickness, Cortical thickness, RIs, YMs). Association analysis was conducted with Spearman’s rank correlation coefficient.

## 3. Results

**Figure 3:**
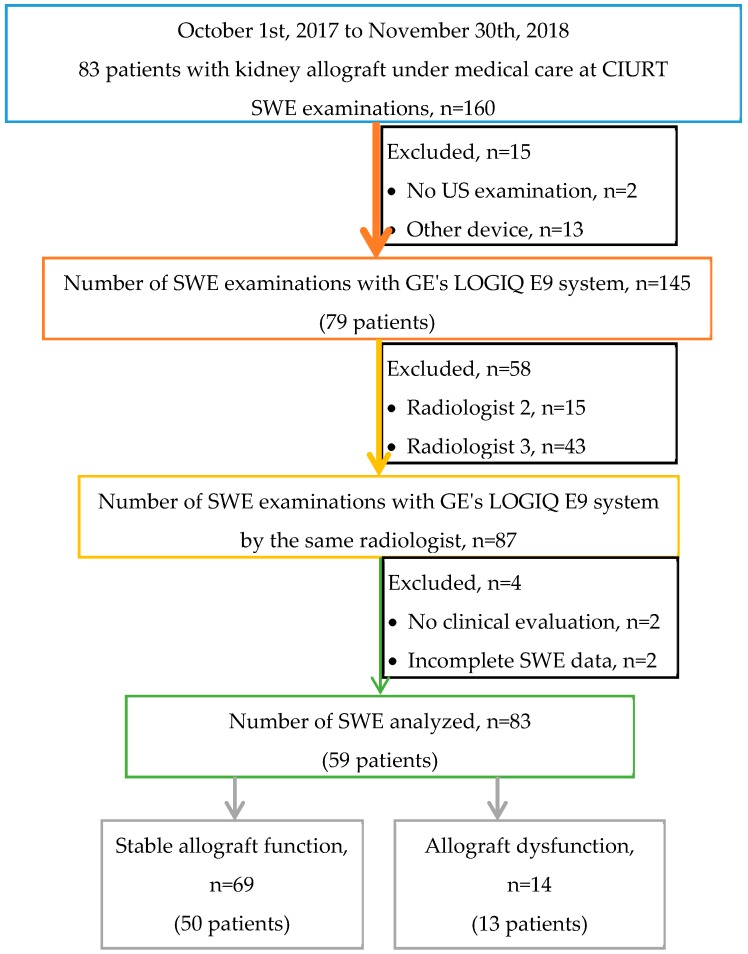
Diagram illustrating the flow of the study, from the eligible population to the inclusion of SWE examinations in the analysis. The characterization of the kidney allograft function was done according to clinical evaluation and laboratory measurements. Nineteen patients with stable allograft have two SWE evaluations; one patient with allograft dysfunction had two examinations.

Eighty-three patients with a kidney transplant were evaluated during the period of the study, and all agreed to participate in the study, cumulating 160 examinations. Eighty-three SWE examinations carried out by the same radiologist with a LOGIQ E9 device belonging to 59 patients were included in the analysis (Figure 3).

The main characteristics of the patients included in the study are given in Table 1.

The body mass index of women (median = 23.5 kg/m^2^, IQR = (22–24.75)) had significantly lower values as compared to men (median = 26 kg/m^2^, IQR = (23–30); Mann-Whitney test: Z-stat = −2.11, *p*-value = 0.0350). Moreover, a significantly higher percentage of women had normal weight (72.7%) while in most of the cases the men were overweight (35.1%) or obese (29.7%) (Chi-square test: Χ^2^ = 8.01, *p*-value = 0.0182).

Glomerulonephritis (32 patients, 54.2%) was the main causes of chronic kidney disease (CKD) in our cohort, followed by CKD with unknown etiology (nine patients, 15.3%), pyelonephritis (7 patients, 11.9%), polycystic diseases (seven patients, 11.9%), and other causes (four patients, 6.8%).

The histopathological examination revealed one case with an inconclusive result, one case with pyelonephritis, and one case with no specific histological features. All other cases were with acute cellular rejection (eight cases) or chronic humoral rejection (two cases).

The age of patients at the SWE examination ranged from 24 to 64 years, with no difference between the group with clinical dysfunction and those with stable allograft (Mann-Whitney test: Z-stat = 1.40, *p*-value = 0.1619). The time from transplant to SWE examination ranged from 30 days to 4279 days with significantly higher values for patients with allograft dysfunction (median = 1668 days (753–2078)) as compared to those with stable function (median = 635 days (277–807), Mann-Whitney test: Z-stat = 3.78, *p*-value = 0.0002).

As expected, significant differences in serum creatinine levels, eGRF, Proteinuria/Creatinuria ratio were observed between cases with allograft dysfunction as compared to those with stable function (Table 2). The percentage with suboptimal dose of calcineurin inhibitor in cases of allograft dysfunction was significantly higher (Table 2).

The time from transplant to examination (Mann-Whitney test: Z-stat = 3.27, *p*-value = 0.0011), serum creatinine level (Mann–Whitney test: Z-stat = 4.51, *p*-value < 0.0001) and Proteinuria/Creatinuria ratio (Mann–Whitney test: Z-stat = 3.67, *p*-value = 0.0002) had significantly higher values in cases with biopsy-proven kidney allograft dysfunction compared to the rest, while eGRF proved to be significantly lower (Mann–Whitney test: Z-stat = −4.42, *p*-value < 0.0001).

Except for echogenicity, no significant differences were observed regarding the morphology or vascularity measured by the resistivity index of the kidney allograft when cases with clinical dysfunction were compared to those with stable function (Table 3).

The resistivity index exceeded the value of 0.8 in three cases for the upper renal artery (one case with clinical allograft dysfunction), four cases for the middle renal artery (one case with clinical allograft dysfunction) and five cases for lower renal artery (one case with clinical allograft dysfunction).

The values of YM showed high variability within the kidney zone and between patients (Table 4). The CQV per examination was under 15% in 54 cases for the kidney cortex (65.1%) and in 27 cases for kidney medulla (32.5%).

The intraclass correlation coefficient of YM was 0.725 (95% CI (0.520–0.823), *p*-value< 0.0001) for kidney allograft cortex (inter–measurements correlation of 0.850, 95% CI (0.807–0.896) for first zone and of 0.786, 95% CI (0.708–0.843) for the second zone). The intraclass correlation coefficient of YM was 0.656 (95% CI (0.540–0.742), *p*-value < 0.0001) for measurements at the level of kidney medulla (inter–measurements correlation of 0.725, 95% CI (0.533–0.822) for the first zone and respectively equal to 0.750, 95% CI (0.679–0.804) for the second zone).

**Figure 4:**
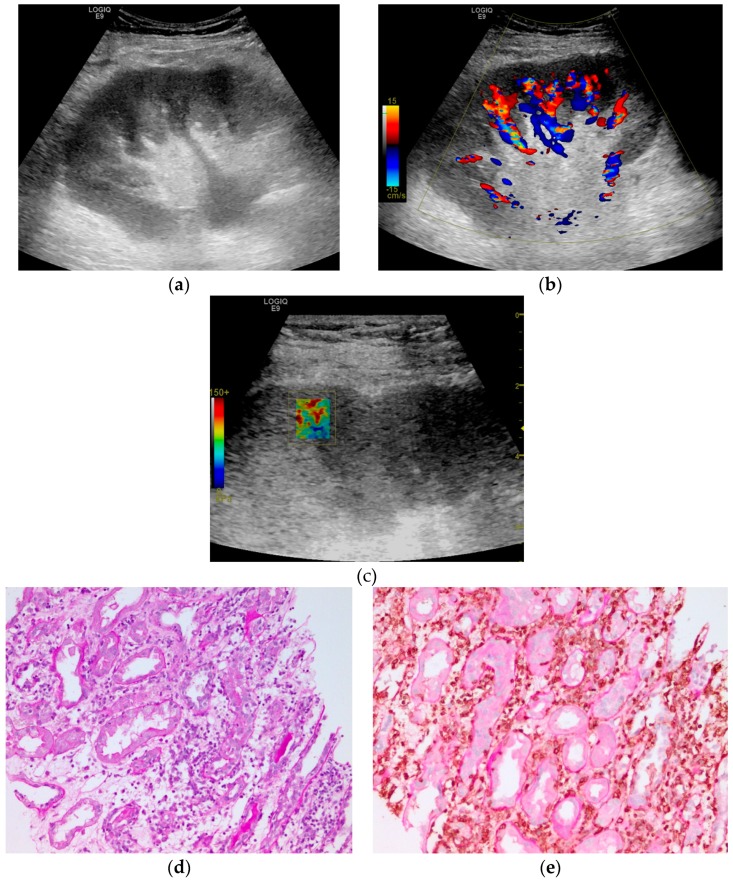
Ultrasound evaluation of a male patient, 62 years old, overweight, evaluated at five years after transplant. The patient was with clinical allograft dysfunction, with eGFR of 12 mL/min/1.73 m^2^ and normal dose of calcineurin inhibitor. The allograft volume was 397 mL, with a parenchymal thickness of 27.1 mm and cortical thickness of 18.1 mm, high cortical–medullar differentiation and grade I hydronephrosis (**a**). Doppler signal was globally present (**b**) with all RIs (RI = resistivity index) higher than 0.8. The SWE elastogram box showed a mild heterogeneous pattern: blue-greenish background with red spot inclusion (**c**) with a median YM equal with 26.6 kPa for cortex and 10.6 kPa for medulla and high intra-patient YM heterogeneity (CQV = 30.2% for cortex and 15.6% for medulla). Microscopic examination reveals severe tubulitis in a background of interstitial edema (**d**) and inflammation (marked brown at CD45 immunostaining over PAS–Periodic Acid–Schiff–counterstaining, 20×); (**e**). The histopathological diagnostic was cellular rejection Banff IB.

**Figure 5:**
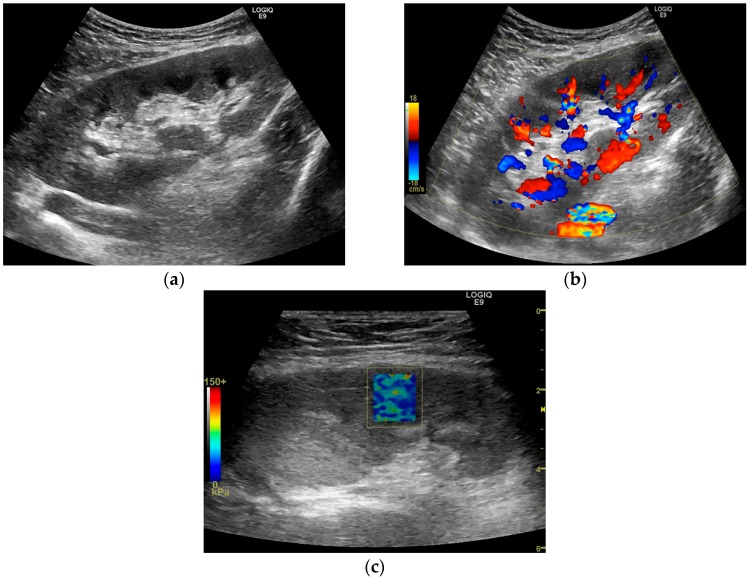
Ultrasound evaluation of a male patient, 27 years old, with normal weight, evaluated at 2005 days after transplant. The kidney allograft function was stable, and the patient was with normal dose of calcineurin inhibitor. The allograft volume was 245 mL, a parenchymal thickness of 12.9 mm, a cortical thickness of 6.3 mm, and normal echogenicity (**a**). Doppler signal was globally present (**b**) with all RIs (RI = resistivity index) lower than 0.7 (0.51 at upper interlobar artery, 0.63 at the medium interlobar artery, and 0.56 at the lower interlobar artery). The SWE elastogram box show homogenous colors around low shiftiness indicating the presence of stiff tissue (**c**) with a median value of YM equal with 37.27 kPa for kidney cortex and 14.31 kPa for kidney medulla and a low variability among measurements on both kidney compartments (QCV equal with 11.13% for cortex and 7.36% for medulla).

The YM at the kidney allograft medulla had significantly higher values in cases with dysfunction (median = 28.70 kPa, (25.68–31.98) kPa) as compared to the cases with stable kidney allograft function (median = 20.99 kPa, (16.08–27.68) kPa; Mann–Whitney test: Z–stat = 2.45, *p*-value = 0.0142). A similar difference was also observed when the cases with clinical kidney dysfunction were compared to those with stable kidney allograft function (Mann–Whitney test: Z–stat = 2.23, *p*-value = 0.0257). A case of kidney allograft dysfunction is presented in Figure 4. Figure 5 presents a case of a patient with a stable kidney allograft function.

The YM had higher values on kidney allograft cortex (median = 21.94 kPa (16.26–28.49)) as compared to medulla (median = 11.24 kPa (8.13–15.67)), and the difference remained regardless of the presence of dysfunction (standard diagnostic-biopsy: median = 28.70 kPa (25.68–31.98) kidney cortex vs. median = 14.40 kPa (11.76–17.20) kidney medulla; vs. clinical diagnostic: median = 27.82 kPa (24.92–31.67) kidney cortex vs. median = 14.40 kPa (11.20–17.34) kidney medulla) or the absence of dysfunction (standard diagnostic: median = 20.99 kPa (16.08–27.68) kidney cortex vs. median = 10.88 kPa (8.03–15.13) kidney medulla; vs. clinical diagnostic: median = 20.99 kPa (16.01–27.66) kidney cortex vs. median = 10.88 kPa (8.05–14.88) kidney medulla).

Only body mass index, time from transplant to SWE examination, and Proteinuria/Creatinuria ratio were significantly associated with the elasticity evaluated by YM, but remained statistically significant just in patients with stable kidney allograft (Table 5).

## 4. Discussion

Kidney parenchymal stiffness measured by shear-wave elastography showed variability in both the intra- and inter-examinations. Parenchymal stiffness showed higher values at the kidney allograft cortex as compared to the medulla Furthermore, significantly higher cortex stiffness was observed in patients with allograft dysfunction as compared to those with stable function.

The most common cause of graft failure beyond the first year after a kidney transplant is represented by nephropathy observed in 24.7% of recipients in the first year with a yearly increase up to 89.9% of the recipients at ten years after transplantation [34]. Early detection of allograft dysfunction is essential, and changes in blood creatinine levels, eGRF, and Doppler-RI are the main used follow-up metrics [35,36]. Shear wave elastography is considered potentially suitable elastography method for the assessment of fibrosis in kidney recipients, but no examination protocol or reference value had yet established [37]. A small amount of evidence relating to SWE for kidney transplant evaluation exists in the scientific literature, showing higher parenchymal stiffness in kidney allograft as compared to stable function [22,23], association with fibrosis [19], significantly lower stiffness values in overweight or obese patients [37]. We conducted this study to examine the intra- and inter-examinations variability of kidney allografts SWE stiffness as an additional tool in post kidney transplantation evaluation. The patients included in our cohort had similar demographic characteristics in terms of age and gender with the patients investigated by Ma et al. [23], but are younger as compared to those investigates by Järv et al. [37].

The investigated cohort showed similar characteristics of patient with kidney allograft dysfunction as those with stable function regarding the age of recipient (*p*-value > 0.10) but with a higher percentage of suboptimal dosage of calcineurin inhibitors (Table 2). This result is explained by the minimization of immunosuppression in order to avoid toxicity/patient non-adherence. The presence of significantly higher values of serum creatinine level and Proteinuria/Creatinuria ratio observed on patients with the gold standard diagnostic of kidney dysfunction compared to those with stable function (*p*-values < 0.0003) sustain the accuracy of the applied clinical classification of allograft dysfunction.

The presence of similar morphological and functional characteristics of the kidney allografts among those with dysfunction as compared to those with stable function indicate no feature characteristics among these two groups (Table 3). However, in our cohort, the values of RIs exceeded 0.8 in only three cases with kidney allograft dysfunction and value higher than 0.8 were observed in seven cases with stable function. Would the patients with RIs > 0.8 develop a kidney allograft dysfunction? The long-term follow-up of these patients will provide an answer to this question. However, other authors reported higher RI values for patients with acute rejection (0.77 ± 0.11) as compared to those with stable function (0.71 ± 0.11) [38]. Similar to our result, Köhnke et al. showed low sensitivity and low specificity of RI in the identification of acute renal allograft rejection [7]. Meier et al. introduce the serial duplex index (SDI = [(RI_t0_/RI_t-1_) × (PI_t0_/Pi_t-1_)]/(CPP_t0_/CPP_t-1_), were CPP = cortex-pelvis proportion, PI = pulsatility index, t0 = values from the day of biopsy, t-1 = values from three to seven days before biopsy) and investigated how its value varies on normal kidney graft function, acute tubular necrosis, acute cellular rejection and acute vascular rejection [39]. However, age [40], pulse pressure, heart rate and cardiac rhythm [41] affect the RIs values and thus could explain the controversially RI results on kidney allograft evaluation [37,40,42,43,44].

The CQV is a measure of spread that depends on the estimation of location (first and third quartile in our study) and is used in laboratory medicine to assess the precision of measurements. An inter- and intra-assay CQV under 15% is considered acceptable [45,46], and CQV small values are characteristics for a reliable method [47]. The stiffness measurements in our study showed a wide range of values, from intra-examination homogeneity (2.21%) to intra-examination heterogeneity (45.04%) at the level of both renal cortex and medulla. The SWE measurements also showed high inter-examination variability (Table 4). However, the intraclass correlation coefficient of YM showed consistency between the ten measurements per cortex and respectively 10 measurements per medulla with slightly higher values for the cortex, but with correlations less than 0.8. The depth of the medulla could explain the observed difference as compared to the kidney allograft cortex, but considering the complexity of the factors that interact with the values, the measurements showed a moderate consistency. However, the CQV threshold for precision in inter- and intra-examination(s) must be determined to properly integrate intra- and inter-patient(s) variability. A larger sample size with dynamic (e.g., before biopsy and on the same day with the biopsy) SWE examination along with the histopathological diagnostic are needed for such study to assure the identification of YMs thresholds able to discriminate the cases with kidney allograft dysfunction by those with stable function. Furthermore, considering the translation of different histopathological changes into the SWE measurements in patients with kidney allografts is also of interest, and our team considers such a study on a larger cohort.

In our study, the SWE stiffness showed significantly higher YM cortex values as compared to the medulla, regardless of the presence or absence of allograft dysfunction (clinical or histopathologic diagnosis). Furthermore, the patients with allograft dysfunction had a significantly higher stiffness at the cortex as compared to those with stable allograft function (*p*-value < 0.02). The values of the tissue stiffness at cortex in our study are similar to those reported by Ma et al. [23], but the medians are slightly higher as compared to the values reported by Ghonge et al. for chronic allograft dysfunction (24.50 kPa ± 4.49 (range, 17.07–32.98 kPa)) [22]. Grenier et al. [48] performed a pilot study and quantified the kidney allograft stiffness using supersonic shear imaging (SSI) [49] that use two spatially extended plane shear waves and consequently enlarge the area of available information. The median cortex stiffness (22.9 kPa) reported by Grenier et al. [48] is similar to values identify in our study for patients with clinical allograft dysfunction but with higher values of medulla stiffness (16 kPa) and small values of coefficient of variability (12% for allograft cortex and 18% for medulla).

The association analysis for tissue stiffness showed a significant association with BMI (negative association) for both cortex and medulla values and Proteinuria/Creatinuria ratio (positive association) (Table 5). Considering the small number of patients with kidney allograft dysfunction, these results need to be validated on larger cohort. Previous studies have reported significant correlation of kidney allograft stiffness with eGRF (negative), RI (positive) and serum creatinine level (positive) (*p*-values < 0.05) [22,50,51]. The positive correlation of parenchymal stiffness with serum creatinine level could reflect the link between allograft function and stiffness, but this relation was not proven in our study, nor in the study conducted by Brocchi et al. [52]. Ma et al. reported significantly (*p*-values < 0.03) positive correlations of cortical (0.26) and medullary stiffness (0.59) with interstitial fibrosis [23]. Järv et al. [37] have also reported the association between the cortex and medulla allograft stiffness and body mass index, with lower mean SWE values on overweight or obese patients (*p*-value = 0.006), a similar result being also found in our cohort.

Several limitations of our study need to be considered. First, this is a single-center study conducted over one-year, and therefore the investigated sample size is limited, and the reported results do not support generalizability or the change of clinical practice in the evaluation of kidney allograft function. Furthermore, the measurement of kidney allograft stiffness is dependent on a series of biological factors such as blood and urinary pressure, the recipient and donor atherosclerosis and age, as well as mechanical factors such as the pressure on the probe, the subjective evaluation of anisotropy in post-processing, etc. The experimental design reduces the possible influence of some above-listed factors (e.g., empty bladder, stenosis, etc.), but the small sample size did not support the correction of such factors during the statistical analysis. The development of a SWE investigation guideline for kidney allografts evaluation and inclusion of more centers over a more extended period will assure a reliable assessment of the stiffness variability as well as validation of the identified results. Second, the function of patients was clinically classified as kidney allograft dysfunction or stable function, and only patients with clinical dysfunction underwent kidney biopsy. Establishing the true thresholds for tissue stiffness requires a gold standard diagnosis, namely allograft biopsy. An experimental design based on the gold standard diagnostic (biopsy) will help establish reliable thresholds of tissue stiffness that allow the identification of allograft dysfunction and classification on different dysfunction grades. Third, the SWE examination was performed by one radiologist during a single sitting, and therefore the interobserver variations could not be determined. Fourth, the investigated cohort was relatively small, most of the transplants were from deceased donors, and the patients were predominately male; thus, generalizability is not recommended. The evaluation of kidney allografts from living and deceased donors, as well as the evaluation of donor features, could also be beneficial in the SWE assessment of kidney allografts.

## 5. Conclusions

The cortical tissue stiffness proved significantly higher values on patients with allograft dysfunction as compared to patients with stable function but to evolve as an additional tool for evaluation of patients with a kidney transplant and change the clinical practice more extensive studies are needed. Kidney tissue stiffness showed high intra- and inter-examination variability, and this variability need to be explained. Extensive studies are needed to identify the kidney allograft stiffness in order to assess the capacity of SWE to discriminate between patients with and without allograft dysfunction.

## Figures and Tables

**Table 1:** Main characteristics of the investigated cohort.

Parameter	Kidney Recipient	Donor
Age at kidney transplant, years	48 (37–55) {23 to 64}	55 (47–62.5) {13 to 72}
Women, no. (%)	22 (37.3)	27 (45.8)
BMI, kg/m^2^	25 (22.5–28.5) {18 to 33}	
BMI classification, no. (%)		
Normal weight	29 (49.2)	
Overweight	17 (28.8)	
Obesity	13 (22.0)	
Mismatch HLA	5 (4–5) {2 to 6}	
Ac-anti-HLA class I, no. (%)	5 (8.5)	
Ac-anti-HLA class II, no. (%)	6 (10.2)	
Alive, no. (%)	10 (16.9)	10 (16.9)
Cause of death, no. (%)		
Stroke	32 (54.2)	32 (54.2)
Craniocerebral trauma	11 (18.6)	11 (18.6)
Suicide	4 (6.8)	4 (6.8)
Cardiorespiratory arrest	2 (3.4)	2 (3.4)

When not specified, data are expressed as median (Q1–Q3) {min to max}.

**Table 2:** Kidney allograft measurements and doses of the calcineurin inhibitor of the investigated cohort.

Parameter	All, *n* = 83	Dysfunction, *n* = 14	Stable Function, *n* = 69	Statistics (*p*-Value)
Serum creatinine level, mg/dL	1.46 (1.19–1.96)	3.71 (2.66–5.10)	1.33 (1.16–1.63)	5.09 (<0.0001)
eGRF (mL/mim/1.73 m^2^)	52 (32–64)	16 (12–30)	54 (43–69)	−4.95 (<0.0001)
Proteinuria/Creatinuria ratio	0.200 (0.106–0.997)	2.145 (1.483–7.603)	0.160 (0.095–0.380)	4.45 (<0.0001)
Calcineurin inhibitor				n.a. (0.0037)
Normal	49 (61.3)	3 (25.0)	46 (67.6)	
Suboptimal dose	15 (18.8)	6 (50.0)	9 (13.2)	
Over dose	16 (20.0)	3 (25.0)	13 (19.1)	

Data are expressed as median (Q1–Q3) for eGRF and Proteinuria/Creatinuria ratio and the groups are compared with the Mann-Whitney test; The dosage of calcineurin inhibitor is expressed as no. (%) and the association with the dysfunction or stable function was tested with Fisher’s exact test. n.a. = not available.

**Table 3:** Grey-scale and Doppler ultrasound characteristics.

Parameter	All, *n* = 83	Dysfunction, *n* = 14	Stable Function, *n* = 69	Stat. (*p*-Value)
Allograft volume, ml	186 (150–226.5)	186 (164.8–247.5)	186 (150–225)	0.27 (0.7844)
Parenchymal thickness (mm)	14.7 (13.2–16.7)	14.1 (13.2–14.6)	15.0 (13.2–16.7)	−1.01 (0.3128)
Cortical thickness (mm)	7.5 (6.6–8.6)	6.9 (5.6–8.4)	7.7 (6.8–8.6)	−1.26 (0.2060)
Echogenicity				n.a. (0.0113)
Normal	65 (78.3)	7 (50.0)	58 (84.1)	
High cortical-medullar diff	14 (16.9)	5 (35.7)	9 (13.0)	
Diffuse hyperechogenicity	4 (4.8)	2 (14.3)	2 (2.9)	
Resistivity Index	0.63	0.60	0.64	−0.86 (0.3913)
Upper interlobar artery	(0.58–0.69)	(0.58–0.66)	(0.58–0.69)	
Resistivity Index	0.66	0.64	0.66	−0.51 (0.6095)
Medium interlobar artery	(0.62–0.70)	(0.59–0.74)	(0.63–0.69)	
Resistivity Index	0.66	0.64	0.66	−0.52 (0.6010)
Lower interlobar artery	(0.62–0.71)	(0.62–0.70)	(0.62–0.71)	

Data are expressed as median (Q1–Q3) (the comparison between groups was done with Mann-Whitney test) excepting the echogenicity were number and (%, association tested with Fisher exact test) were reported; n.a. = not available.

**Table 4:** Variability analysis of Young’s modulus between examinations by clinical classification of kidney allograft as dysfunction or stable function.

	All, *n* = 83		Dysfunction, *n* = 14		Stable Function, *n* = 59	
	Cortex	Medulla	Cortex	Medulla	Cortex	Medulla
A1	34.43 (27.42–41.76)	33.08 (20.19–47.96)	38.19(23.24–64.92)	43.55(24.35–74.75)	30.99 (23.63–39.32)	33.96 (17.93–51.91)
A2	30.99 (23.72–43.10)	42.38 (33.00–53.18)	34.15 (16.94–53.57)	39.19 (17.51–66.43)	28.70 (19.49–41.85)	40.90 (29.01–52.32)
A3	31.98 (24.39–40.99)	33.16 (21.08–45.90)	26.17 (13.01–45.18)	28.91 (7.65–66.16)	31.61 (22.82–40.57)	33.90 (21.24–45.45)
A4	28.66 (23.77–36.15)	40.20 (28.10–49.22)	19.33 (4.50–46.02)	36.59 (11.25–64.63)	28.83 (22.69–38.20)	39.06 (27.74–49.65)
A5	30.37 (22.02–40.49)	34.32 (23.41–45.70)	23.99 (9.84–54.64)	37.72 (20.86–81.13)	29.29 (22.20–40.93)	32.22 (20.25–45.05)
B1	29.78 (24.53–37.89)	37.34 (30.03–45.86)	22.91 (7.40–41.12)	30.95 (11.02–51.05)	27.91 (17.00–35.27)	38.83 (30.89–48.38)
B2	32.00 (26.18–38.84)	36.04 (27.19–43.38)	17.53 (7.64–37.44)	27.09 (15.28–49.70)	29.76 (21.90–35.88)	38.92 (29.92–48.86)
B3	28.91 (24.23–34.36)	40.87 (32.85–50.50)	21.91 (3.85–39.25)	31.63 (12.99–57.28)	28.09 (22.28–34.44)	40.57 (30.72–52.00)
B4	28.79 (23.12–34.20)	31.54 (21.98–40.83)	24.64 (12.52–54.28)	24.81 (8.86–41.99)	25.87 (19.21–32.26)	32.11 (22.08–44.20)
B5	33.9 (26.22–42.93)	39.12 (30.61–48.38)	21.19 (11.69–46.57)	17.59 (1.59–36.54)	32.77 (23.32–41.77)	38.79 (27.13–48.24)
Median	27.34 (21.52–33.01)	30.81 (23.29–41.43)	24.64 (13.20–54.65)	24.81 (11.50–49.53)	26.67 (21.71–35.13)	28.56 (18.07–39.55)

Data are expressed as CQV values and 95% confidence interval (lower bound–upper bound); A is the first zone and B is the second zone with five measurements in each zone for cortex and five for medulla.

**Table 5:** Statistically significant associations between Young’s modulus and demographic and clinical characteristics.

Parameter	All Cohort, *n* = 83	Dysfunction, *n* = 11	Stable Function, *n* = 72
BMI & YM cortex	−0.44 (<0.0001)	−0.47 (0.1412)	−0.39 (0.0008)
BMI & YM medulla	−0.42 (0.0001)	−0.45 (0.1691)	−0.38 (0.0010)
TimeTE & YM cortex	0.36 (0.0008)	0.45 (0.1601)	0.30 (0.0110)
PCR & YM cortex	0.33 (0.0021)	0.05 (0.8734)	0.27 (0.0200)
PCR & YM medulla	0.28 (0.0105)	0.18 (0.5926)	0.24 (0.0436)

BMI = Body Mass Index expressed in kg/m^2^; YM = Youn’s Modulus express in kPa; TE = transplant–to–examination expressed in days; PCR = Proteinuria/Creatinuria ratio.
